# On the Operation for Strabismus

**Published:** 1840-12

**Authors:** 


*On the Operation for Strabismus.* By Professor DIEFFENBACH.—Since my first communication on this operation, it has had such a general reception, and has acquired such an importance as I did not at that time anticipate. Upwards of three hundred cases have been operated on by me within a few months, and both in Berlin and in other places my proceeding has been frequently imitated. I propose to give here a short general view of the results of my observations.

The youngest individuals in whom I have undertaken the division of the shortened muscle of the eye were five years old; the oldest were upwards of forty.

Sometimes one, sometimes both eyes squinted, and the operation had generally the same favourable result in both cases. When both eyes were affected, I either operated first on that which squinted most, and when that was quite well on the other, or else on both at the same time.

Squinting inwards, from shortening of the rectus internus, was by far most frequent. Sometimes the trochlearis muscle was also shortened, so that it was necessary to divide it as well as the rectus. In the whole number of those I operated on there were only a few who squinted outwards, and still fewer in whom the eye was directed upwards, or upwards and inwards. I found no eyes at all that squinted downwards.

Strabismus upwards was sometimes complicated with blepharoptosis. The division of the rectus superior not only cured the squinting, but the ptosis gradually diminished after it.

Strabismus outwards or inwards was often complicated with nystagmus bulbi. After the division of the external or internal rectus, not only did the squinting cease, but in general the nystagmus also. In other cases, however, the latter was persistent, and did not decrease till after the division of the rectus superior, or obliquus superior, or rectus externus.

When cataract and strabismus co-existed, the operations for both were done at the same time, and the result was in every case favourable to both.

In most of the patients the strabismus had commenced in very early childhood after ophthalmia neonatorum, scrofulous inflammation of the eyes with ulcers on the cornea, or after acute exanthemata, &c. In many there were cicatrices on the cornea or cataracta centralis. In cases of the former kind, in which hitherto artificial pupils would have been made, the operation was attended by success and considerable improvement of the sight.

All those who had strabismus of only one eye saw more weakly with it than with the other; in those who squinted with both eyes, that which was turned least, was usually the stronger. The weakness of the one eye had been observed by only a few of the patients; they had naturally looked only with the better eye, and the other had been unemployed. The operation completely cured the weakness of sight; some who had actually amaurotic amblyopia could see clearly directly after it was performed.

Some of the patients, previous to the operation, often saw double; this defect continued for some time after it, and then gradually ceased. Some others who had never seen double before did so immediatelv after the operation. These had been in the habit of looking only with their strong eye while the other had been unused. The improved position of the latter compelled it to see; but the double vision was subsequently lost.

Some who were operated upon did not see so well immediately after as before the operation; but after some exercise this weakness of vision ceased, and they could then see quite clearly. The cause of this was that when the eye was put in its normal position, a point of the retina, which was before unexercised, was now brought into play, and required some practice before it could fully discharge its functions.

*Operation.*—That for strabismus convergens is here taken as the type. The operator always stands on the right side of the patient, whether he be operating on the right or left eye. The patient sits on a stool, and an assistant standing behind him draws up the upper eyelid with a Pellier's hook. A second assistant draws down the lower eyelid with a double hook which is set in a handle, and of which the teeth are connected by a transverse piece. He kneels down before the patient so as not to be in the way.

The operator then puts a fine hook into the conjuctiva, at the inner angle of the eye, just where it is passing from the palpebræ to the bulb, passes it superficially through it, and gives it to a third assistant who stands on the left side of the patient. The operator next passes a second hook in the same way through the conjunctiva about a line and a half from the first. He and his assistant then both at the same time draw their hooks a little up, so as to raise a fold of the conjunctiva, and at the same time pull the bulb somewhat outwards. The fold is then divided with a pair of curved eye-scissors; and this cut usually at once exposes the tendon and the anterior part of the muscle. A couple of cuts with the scissors then expose the outer surface of the muscle; a rather blunt hook is passed under its tendon, and the two sharp hooks that held the conjunctiva are now removed; the eye is held completely in the power of the blunt hook, and is to be drawn by it from out the internal angle of the orbit. A flat probe is then pushed under the muscle; and the loose connexion by cellular tissue between it and the eye is broken up. The division of the muscle is made by the scissors already mentioned, either, first, through the tendon in front of the hook; or, second, behind the hook at the beginning of the muscular substance; or, third, some lines deeper back.

When the tendon is divided nothing of it remains on the eye, and the muscle commonly retracts a line backwards. When the muscle itself is divided at its anterior part or further back, its posterior portion retracts, and the anterior, which remains connected with the bulb, turns forward like a loose flap, which, according to circumstances, may be removed by the scissors, or pushed back into the wound if it is thought desirable that it should unite again with the posterior portion.

In practised hands the whole operation seldom lasts more than a minute; and it is done almost without pain. When finished, the eye is cleaned with cold water and a soft sponge. The after-treatment consists of cold lotions, and very great abstinence from food and strong drinks. The patient should be kept in a darkened room. In most cases the wound heals very quickly; and after a few weeks no traces of the operation remain, and the eye stands in its normal position.

The operation for internal strabismus is by far the most easy; the division of the obliquus superior for squinting upwards and inwards is more difficult; that of the rectus externus for strabismus divergens is more difficult still; and the most difficult of all is the division of the rectus superior for squinting upwards. With respect to the manipulations of these operations, they are just the same as those for Strabismus convergens.

*Remarks on the operation.*—The fixing of the upper and lower eyelids with the elevator and the hook, so as to expose the whole of the anterior surface of the globe, is indispensable; for neither the will of the patient, nor the separation of the lids by the finger, can do this effectually.

The fixing of the globe can be accomplished only by fine hooks carried superficially through the conjunctiva; the seizing and elevation of the fold of conjunctiva by forceps, sounds more gentle than to do it with a sharp hook; but it is in reality far more painful, more injurious, and more insecure; the fold raised up by the forceps easily tears or slips from their grasp, and if the forceps are made with hooks, they wound as well as pinch the membrane. Two hooks must be employed to make the fold tense enough.

The great number of operations that I have performed, has given me opportunity of observing the phenomena that ensue subsequently to them, and their after consequences. The question here is only of internal strabismus, but any surgeon will easily supply the necessary modifications for the operations in the other varieties. In the first case, the eye, after the division of the muscle, goes into its normal position. In the second, it remains in some degree squinting. In the third, it turns outwards.—*Brit. and For. Rev., from Casper's Wochenschrift.*—*Medical Examiner for October.*

